# Draft Genome Sequence of an Obligately Methylotrophic Methanogen, *Methanococcoides methylutens*, Isolated from Marine Sediment

**DOI:** 10.1128/genomeA.01184-14

**Published:** 2014-11-20

**Authors:** Yue Guan, David K. Ngugi, Jochen Blom, Shahjahan Ali, James G. Ferry, Ulrich Stingl

**Affiliations:** a Red Sea Research Center, King Abdullah University of Science and Technology, Thuwal, Saudi Arabia; b Justus-Liebig-Universität Giessen, Bioinformatik und Systembiologie, Giessen, Germany; c Biosciences Core Laboratory, King Abdullah University of Science and Technology, Thuwal, Saudi Arabia; d Department of Biochemistry and Molecular Biology, Pennsylvania State University, University Park, Pennsylvania, USA

## Abstract

*Methanococcoides methylutens*, the type species of the genus *Methanococcoides*, is a slightly halophilic methanogenic archaeon with a methylotrophic metabolism. Here, we present the annotated draft genome sequence of *M. methylutens*, which comprises 2,508,511 bp with 2,482 coding sequences, 51 tRNA genes, and a G+C content of 42.5%.

## GENOME ANNOUNCEMENT

The genus *Methanococcoides* currently comprises four described and characterized species: *M. methylutens* (1), *M. burtonii* (2), *M. alaskense* (3), and *M. vulcani* (4). *M. methylutens* is the type species of this genus that was isolated from submarine canyon sediments off the coast of southern California (1). Together with several other strains closely related to *M. methylutens*, that is, MM1 (5), NaT1 (6), MO-MCD (7), AM1, DM1, NM1, PM1, and PM2 (8) (with 16S rRNA gene identities of 98% to 99%), *Methanococcoides* species have been cultivated from anoxic hypolimnion of Ace Lake and various sediment environments such as marine, mangrove, and mud volcanoes.

To date, only the genome sequence of *M. burtonii* is available (9). Both *M. burtonii* and *M. methylutens* utilize methanol and mono-, di-, and tri-methylamine for methanogenesis and growth but not H_2_/CO_2_, formate, or acetate (1). They have recently been shown to metabolize also *N*,*N*-dimethylethanolamine but not choline or glycine betaine (4, 8, 10). Their 16S rRNA genes share a sequence identity of approximately 98%. *M. burtonii* is psychrophilic and motile, and therefore well distinguished from *M. methylutens*. Here, we announce the genome sequence of *M. methylutens* as a basis for future comparative studies aimed at understanding the ecological niche of this genus.

Genomic DNA of *M. methylutens* DSM 2657 was provided by DSMZ (German Collection of Microorganisms and Cell Cultures). The sequencing library was prepared using the Illumina TruSeq DNA Sample Preparation kit. Sequencing was done using the MiSeq platform in the Bioscience Core Laboratory at King Abdullah University of Science and Technology, generating a total of 11.7 million paired-end reads (mean length 297 bp). The reads were quality filtered, trimmed, and assembled into contigs using the *de novo* assembler SPAdes version 2.5.1 (11).

The draft genome comprises 15 contigs with a total length of 2,508,511 bp (*N*_50_: 532.3 kbp) and a G+C content of 42.5%. Putative coding sequences (CDSs) were predicted using the automated annotation INDIGO pipeline (12) and the NCBI PGAAP annotation service. Of the 2,482 predicted CDSs in the genome, most were homologous to *M. burtonii* genes (~80%). The genome of *M. burtonii* is predicted to encode all enzymes required for methylotrophic methanogenesis and for the oxidation of methyl-coenzyme M through the reverse CO_2_ reduction pathway. Except for Cytochrome *b* (VhoC), the large and small subunits of F_420_-nonreducing hydrogenase (VhoA and VhoG) are present in this genome (absent in *M. burtonii*). Other hydrogenases related to growth with hydrogen and enzymes converting acetate to acetyl-CoA are absent. Genes for dealing with osmotic stress are present in the genome and include genes for the uptake of glycine betaine/ proline and for the biosynthesis of glycogen, glutamate and N^ε^-acetyl-β-lysine. Surprisingly, although *M. methylutens* cells appear nonmotile and corresponding structures were not observed in the investigated culture conditions (1), this draft genome is predicted to encode enzymes for flagellar assembly and chemotaxis.

### Nucleotide sequence accession number.

This whole-genome shotgun project has been deposited at DDBJ/EMBL/GenBank under the accession number JRHO00000000. The version described here is the first version.
